# E2F1 interactions with hHR23A inhibit its degradation and promote DNA repair

**DOI:** 10.18632/oncotarget.8362

**Published:** 2016-03-25

**Authors:** Randeep K. Singh, Lina Dagnino

**Affiliations:** ^1^ Department of Physiology and Pharmacology, Children's Health Research Institute and Lawson Health Research Institute, The University of Western Ontario, London, Ontario, N6A 5C1, Canada

**Keywords:** keratinocytes, epidermis, E2F1, hHR23, DNA photodamage

## Abstract

Nucleotide excision repair (NER) is a major mechanism for removal of DNA lesions induced by exposure to UV radiation in the epidermis. Recognition of damaged DNA sites is the initial step in their repair, and requires multiprotein complexes that contain XPC and hHR23 proteins, or their orthologues. A variety of transcription factors are also involved in NER, including E2F1. In epidermal keratinocytes, UV exposure induces E2F1 phosphorylation, which allows it to recruit various NER factors to sites of DNA damage. However, the relationship between E2F1 and hHR23 proteins vis-à-vis NER has remained unexplored. We now show that E2F1 and hHR23 proteins can interact, and this interaction stabilizes E2F1, inhibiting its proteasomal degradation. Reciprocally, E2F1 regulates hHR23A subcellular localization, recruiting it to sites of DNA photodamage. As a result, E2F1 and hHR23A enhance DNA repair following exposure to UV radiation, contributing to genomic stability in the epidermis.

## INTRODUCTION

Nonmelanoma skin carcinomas are the most frequent malignancies in humans, accounting for about 40% of all diagnosed cancers in North America [1]. Alarming increases in their frequency in the last two decades, particularly in young adults, are associated with increased exposure to UV radiation, which is the primary risk factor [2]. Two major forms of nonmelanoma skin tumours occur, basal cell and squamous cell carcinoma, both of which arise from the transformation of epidermal keratinocytes [1]. Increased risk of developing nonmelanoma skin carcinoma also occurs in individuals affected by xeroderma pigmentosum, an autosomal recessive genetic disorder that arises from an impaired ability of epidermal cells to repair damaged DNA [3].

DNA damage caused by UV radiation is characterized by the formation of cyclobutane pyrimidine dimers (CPD) and pyrimidine (6-4) pyrimidone photoproducts [4]. The activation of nucleotide excision repair (NER) pathways repairs photodamaged DNA, through processes that involve initial recognition of the DNA distortion, followed by excision of the damaged bases and synthesis of new DNA. In human cells, complexes containing the DNA damage recognition factor XPC bound to hHR23A or hHR23B associate with DNA photolesions to initiate NER [4, 5]. A major role for hHR23 proteins during NER appears to involve protection of XPC from proteasomal degradation, thus enhancing NER efficiency.

hHR23 proteins and their orthologues are scaffolds necessary for normal DNA repair, but are also involved in modulating the proteasomal degradation of a variety of proteins [5]. They are modular proteins composed of an N-terminal ubiquitin-like (UbL) domain and two ubiquitin-associated domains (UBA1 and UBA2), which bind ubiquitin or ubiquitylated proteins. Linking the UBA1 and UBA2 regions is the XPC domain, which mediates association of hHR23 with a variety of proteins, including XPC [6]. Depending on the cellular context, binding of hHR23 proteins to ubiquitylated substrates may increase or decrease their proteasomal degradation. hHR23 can bind to and escort polyubiquitylated proteins to the proteasome, increasing their degradation. Paradoxically, hHR23 proteins have also been found to protect XPC and other proteins from proteasomal degradation, possibly by interfering with proteolysis of ubiquitylated XPC by the proteasome [7, 8].

DNA damage triggers upregulation of several proteins, including the E2F1 transcription factor. As a consequence of genotoxic stress, E2F1 is phosphorylated on Ser31 [9]. This modification interferes with E2F1 ubiquitylation and subsequent proteasomal degradation, and is required for E2F1 association with damaged DNA [10]. In response to UV radiation, E2F1 associates with damaged DNA sites, where it recruits additional DNA repair factors, such as GCN5 [11]. In this manner, E2F1 contributes to efficient NER. Important biological consequences of these properties of E2F1 are the maintenance of genomic stability and suppression of tumour development in the epidermis [10].

The well established importance for NER of E2F1 and hHR23, the ability of the latter to modulate proteasomal degradation, and the stabilization of E2F1 upon DNA damage prompted us to investigate if these two factors work together in DNA repair processes. We now show that hHR23 proteins directly associate with and stabilize E2F1. In turn, E2F1 can recruit hHR23A to regions of DNA photodamage, resulting in enhanced DNA repair.

## RESULTS

### Interaction of hHR23A and E2F1

**Figure 1:**
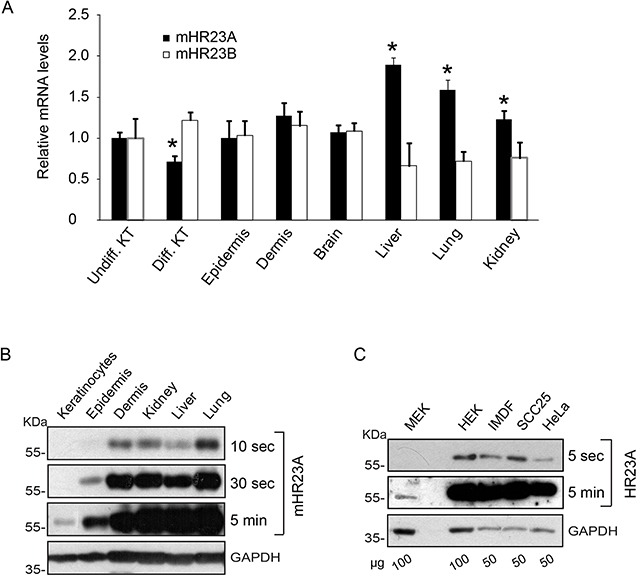
Expression of mHR23 and hHR23 proteins **A.** mRNA was isolated from primary undifferentiated (Undiff. KT) or differentiated (Diff. KT) 3-day keratinocyte cultures, or from the indicated tissues harvested from 2-d-old CD-1 mice. *mHR23A* and *mHR23B* mRNA levels were assessed by qPCR, and normalized to the abundance of *Rpl16* and *Rps29* transcripts. The results are expressed as the mean + SEM (n=3), relative to mRNA abundance in undifferentiated keratinocytes, which is set to 1.0. The asterisks indicate P<0.05, relative to abundance in undifferentiated keratinocytes (ANOVA). **B, C.** The abundance of mHR23A or hHR23A was determined in protein lysates from the tissues harvested in (A) or from the indicated cultured cells. Glyceraldehyde-3-phosphatase (GAPDH) was used to normalize for protein loading. MEK and HEK indicate, respectively, primary cultures of undifferentiated mouse and human epidermal keratinocytes. Blot exposure times and the amount of protein on the blots (in μg) are indicated, to better illustrate differences in protein abundance.

The mammalian orthologues of yeast Rad23 (HR23) proteins play dual roles in DNA NER and modulation of ubiquitin-mediated protein degradation [5]. To begin to address the role of these proteins in the epidermis, we first assessed their expression levels in mouse tissues. Quantitative polymerase chain reaction (qPCR) analysis revealed the presence of transcripts encoding mHR23A and mHR23B in the epidermis and the dermis, at levels comparable to those found in a variety of other tissues (Figure 1A). Further, *mHR23A* and *mHR23B* mRNA species were also detected in primary cultured epidermal keratinocytes, irrespective of whether they were undifferentiated or had been induced to differentiate (Figure 1A). mHR23A protein was detected at low levels in mouse epidermis and in cultured keratinocytes, whereas it was substantially more abundant in various human epithelial cell types and mouse tissues, including the dermis (Figure 1B, 1C).

**Figure 2:**
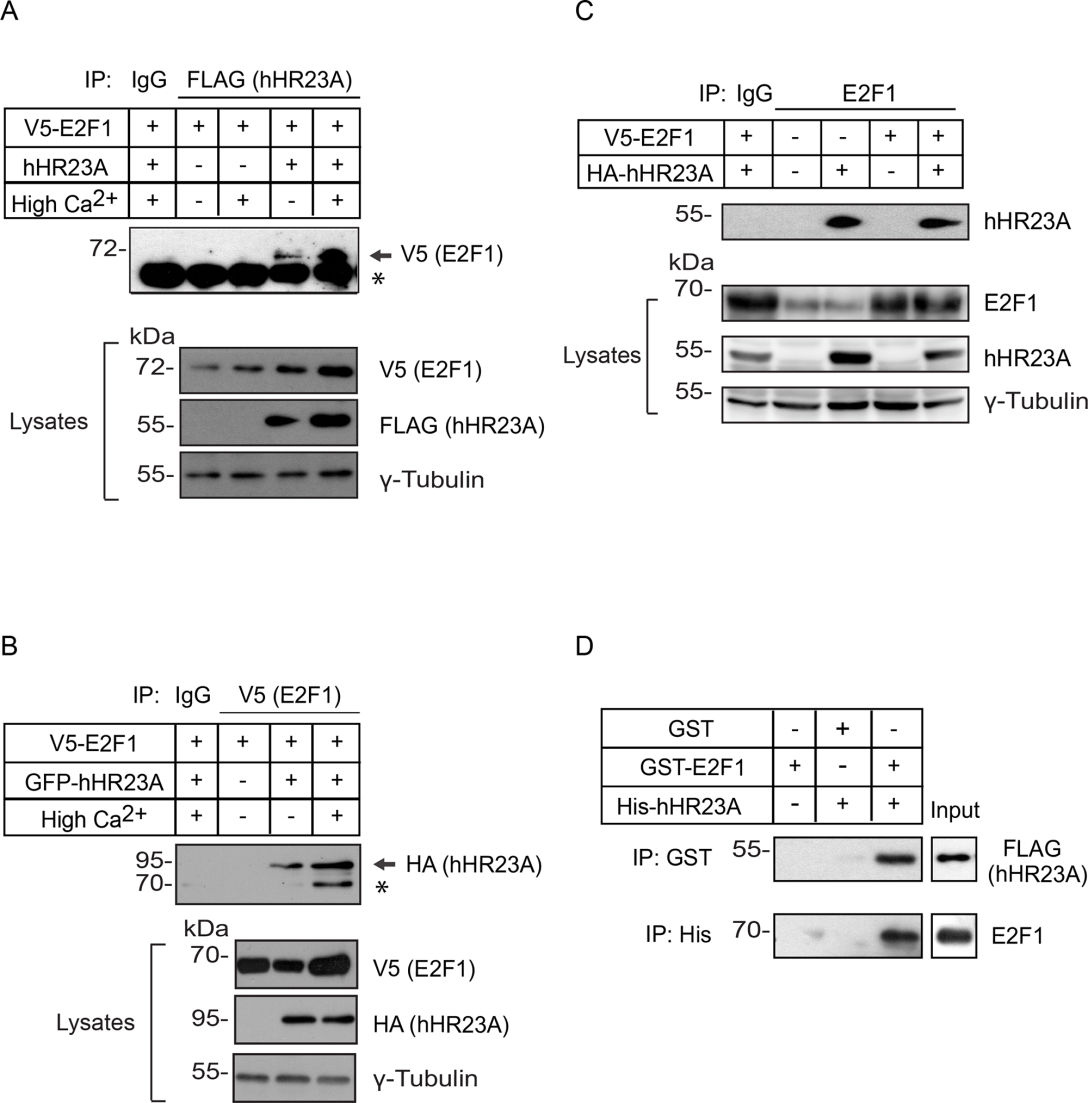
Association of hHR23A and E2F1 **A, B.** Primary keratinocytes were transfected with vectors encoding V5-tagged E2F1 with or without FLAG-tagged hHR23A or HA- and GFP-tagged hHR23A and cultured for 24 h after transfection in Low Ca^2+^ or in High Ca^2+^ medium, to induce differentiation. Cell lysates were prepared and immunoprecipitated with anti-FLAG or anti-V5 antibodies, as indicated, or an irrelevant IgG. Immune complexes were resolved by denaturing gel electrophoresis, transferred to membranes, and the blots were probed with the indicated antibodies. Samples of lysates used for immunoprecipitation show expression levels of exogenous proteins. γ-Tubulin was used to normalize for protein loading, and the asterisks indicate a non-specific band. **C.** Lysates prepared from transfected keratinocytes as in (A, B), were immunoprecipitated with anti-E2F1 antibodies, to isolate endogenous and/or exogenous E2F1 immune complexes. Replicate lysate samples were also analyzed to show expression levels of endogenous and exogenous proteins. **D.** Bacterially produced GST, GST-E2F1, as well as His- and FLAG-tagged hHR23A (2 μg each) were used in immunoprecipitation experiments with anti-GST or anti-His antibodies, as indicated. The immune complexes were further analyzed by immunoblot, using anti-FLAG or anti-E2F1 antibodies. The lanes labelled “Input” contain 100 ng each of the indicated recombinant proteins.

hHR23A and mHR23A proteins participate in NER processes through the formation of multiprotein complexes that recognize sites of DNA damage [5]. The ability of the E2F1 transcription factor to recognize sites of DNA damage induced by UV radiation and to promote NER [12] prompted us to investigate the possibility that E2F1 and hHR23A might associate. To this end, we exogenously expressed V5-tagged E2F1 together with FLAG-tagged hHR23A in primary mouse keratinocytes that had been maintained undifferentiated, or had been induced to differentiate by 24 h of culture in medium containing 1 mM Ca^2+^ (High-Ca^2+^ medium). When we isolated hHR23A immune complexes, we were also able to detect V5-tagged E2F1, irrespective of the differentiation status of the keratinocytes (Figure 2A). Reciprocally, we readily detected hHR23A fused to green fluorescent protein (GFP) and hemagglutinin (HA) tags in V5-E2F1 immune complexes (Figure 2B). The larger molecular mass of the GFP-tagged hHR23A species allowed us to avoid interference of IgG heavy chains in this analysis. We also found that endogenous E2F1 could associate with hHR23A proteins (Figure 2C), but were unable to detect mHR23A in E2F1 immune complexes, likely due to its very low abundance in primary keratinocytes.

hHR23 proteins can function as scaffolds between ubiquitylated substrates and the proteasome. To determine if hHR23A interactions with E2F1 required ubiquitylation of the latter, we examined if bacterially produced hHR23A and glutathione (GST)-tagged E2F1 were able to interact. We readily detected hHR23A in GST-E2F1 immune complexes, indicating that interactions between these two proteins do not require the presence of post-translational modifications (Figure 2D). These observations do not exclude the possibility that binding of hHR23A to E2F1 also occurs through ubiquitylated residues on the latter.

### Modulation of hHR23A subcellular localization by E2F1

**Figure 3:**
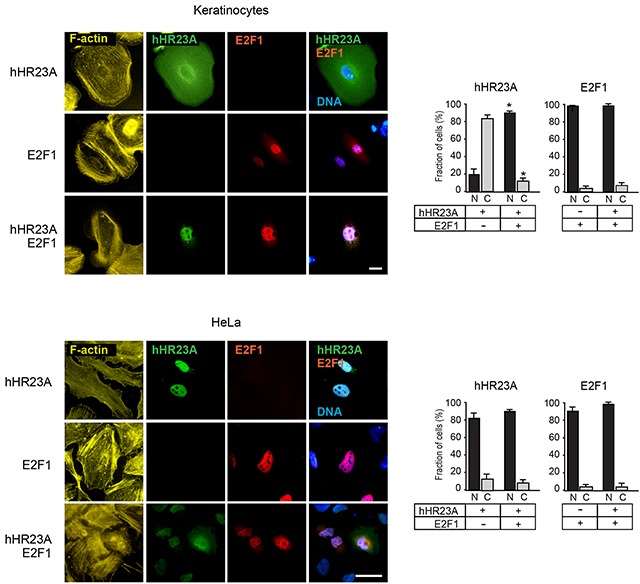
Modulation of hHR23A subcellular distribution by E2F1 Primary keratinocytes or HeLa cells were transfected with vectors encoding the indicated proteins, and 24 h later were processed for immunofluorescence microscopy using the indicated antibodies. The histograms represent the average fraction of cells with nuclear (N) or cytoplasmic (C) protein distribution + SEM (n=4). At least 100 transfected cells per condition were scored in each experiment. The asterisks indicate P<0.05, relative to cells transfected with single vectors (ANOVA). F-actin and DNA were visualized, respectively, with phalloidin and Hoescht 33342. Bar, 25 μm.

Given that hHR23 proteins and their orthologues are involved in both proteasomal degradation and DNA repair, we next investigated the subcellular localization of exogenously expressed hHR23A in undifferentiated keratinocytes. We observed that hHR23A exhibited cytoplasmic distribution in about 90% of the cells (Figure 3). This pattern markedly differs from that observed in transformed HeLa cells, which show nuclear concentration of hHR23A (Figure 3).

In many cell types, including undifferentiated keratinocytes, E2F1 is predominantly found in the nucleus, although it exhibits nucleocytoplasmic shuttling [13] (Figure 3). Significantly, the joint expression of hHR23A and E2F1 resulted in the co-localization of both proteins in the nucleus of almost all keratinocytes (Figure 3). Induction of differentiation in keratinocytes promotes E2F1 nuclear export and degradation [14]. Thus, it was of interest to investigate if the presence of cytoplasmic E2F1 in these cells was associated with loss of nuclear pools of hHR23A. We cultured keratinocytes in medium containing either 0.12 mM or 1.0 mM Ca^2+^, and found that in both culture conditions, when E2F1 was cytoplasmic, hHR23A was also excluded from the nucleus (Supplementary Figure S1). These observations are consistent with the concept that E2F1 is capable of modulating hHR23A subcellular localization and recruitment into the nucleus, possibly mediated via complex formation between these two proteins.

### hHR23A domains involved in binding to E2F1

**Figure 4:**
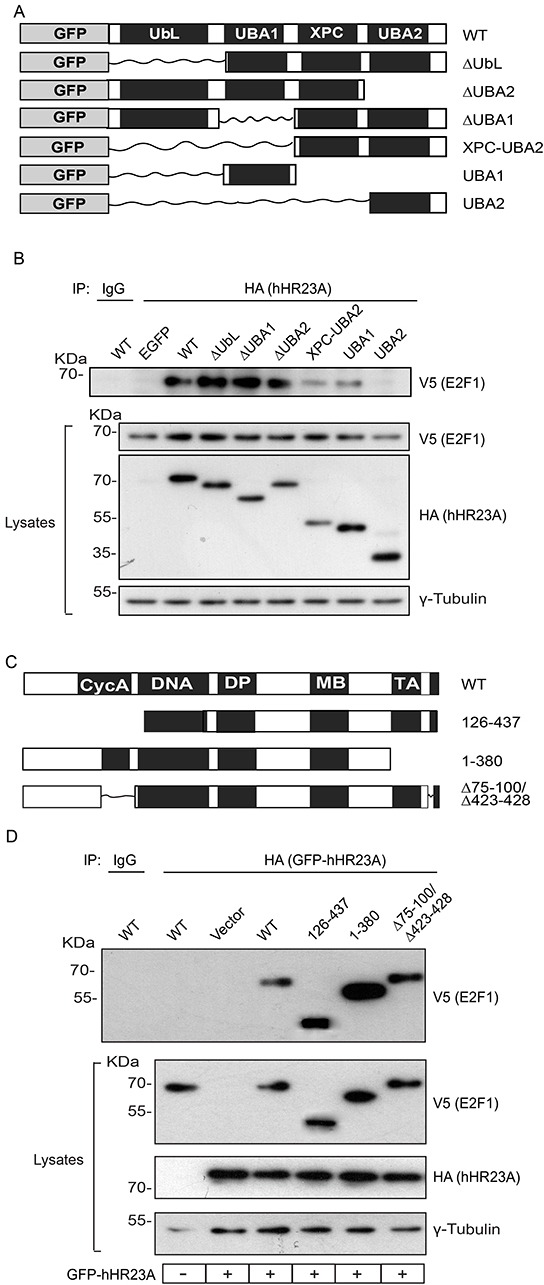
hHR23A domains involved in E2F1 binding **A.** Schematic of GFP- and HA- doubly tagged hHR23A proteins analyzed. **B.** Primary keratinocytes were transfected with vectors encoding V5-tagged E2F1 and the indicated hHR23A proteins. Twenty-four hours after transfection, cell lysates were prepared and hHR23A immune complexes were isolated and analyzed by immunoblot with the indicated antibodies, and γ-tubulin was used to normalize for protein loading. A portion of the lysates from cells co-expressing wild type (WT) hHR23A and V5-tagged E2F1 were also used for immunoprecipitation with an unrelated IgG control. **C.** Schematic of V5-tagged E2F1 proteins analyzed. **D.** Primary keratinocytes were transfected with vectors encoding wild type hHR23A and the indicated V5-tagged E2F1 proteins and analyzed as in (B). A portion of the lysates from cells co-expressing wild type GFP-hHR23A and wild type (WT) V5-tagged E2F1 were also used for immunoprecipitation with an unrelated IgG control.

To begin to understand the biological significance of E2F1 binding to hHR23A, we next undertook an analysis of the protein domains involved in these interactions. As a scaffold protein, hHR23A has several functionally important domains that mediate interactions with ubiquitin and/or various ubiquitin-unrelated proteins [5]. To identify which of those regions are important for E2F1 binding, we exogenously expressed several GFP- and HA-tagged hHR23A deletion mutants (Figure 4A). The use of GFP-tagged hHR23A allowed us to confirm that the GFP moiety did not affect the ability of hHR23A to bind E2F1 (Figure 4B), and allowed us to efficiently express small hHR23A domains for analysis. The N-terminal ubiquitin-like domain (UbL) in hHR23A mediates binding to the proteasome, and is important for protein-protein interactions. We found that deletion of this domain (ΔUbL hHR23A) did not affect the ability of hHR23A to interact with E2F1 (Figure 4B). hHR23A has two ubiquitin-associated domains, UBA1 and UBA2, which mediate interactions with ubiquitin moieties conjugated to various proteins. When we exogenously expressed two hHR23A fragments corresponding to UBA1 or UBA2, we observed that only the former was able to associate with E2F1 (Figure 4B). Thus, the contribution of UBA2 to hHR23A binding to E2F1 is likely negligible, if any. In a complementary approach, we investigated the consequences of deleting UBA1 or UBA2 in the context of the entire hHR23A protein (hHR23A ΔUBA1 or ΔUBA2), and observed efficient binding of these mutants to E2F1 (Figure 4B). This suggests that, in addition to UBA1, other hHR23A regions likely contribute to its association with E2F1. hHR23 proteins bind the xeroderma pigmentosum complementation group C protein (XPC) via their XPC domain [5]. To determine if the XPC region contributes to E2F1 binding, we expressed an hHR23A mutant composed of only the XPC and UBA2 domains (hHR23A XPC-UBA2). We reasoned that this mutant would provide information on the role of the XPC domain in this process, given our previous observation that UBA2 does not bind E2F1.We found that this hHR23A form was still able to bind E2F1 fairly efficiently. However, the XPC region appears to be sufficient, but not indispensable for E2F1 binding, given that an hHR23A mutant lacking these sequences (ΔXPC) also associates with E2F1 (Figure 4B, Supplementary Figure S2). Together, our observations implicate the UBA1 and XPC domains in the association of hHR23A with E2F1.

We undertook a similar analysis to determine the regions in E2F1 involved in binding to hHR23A. The N-terminal domain in E2F1 mediates protein-protein interactions, such as binding to cyclin A, and contains the nuclear localization and export domains responsible for nucleocytoplasmic shuttling [13]. A mutant, constitutively cytoplasmic E2F1 protein lacking these sequences (E2F1 126-437) associated with hHR23A (Figure 4C, 4D), indicating that nuclear localization of E2F1 is not required for this interaction. The C-terminus in E2F1 contains the transcriptional activation domain, and mediates protein-protein interactions with other proteins, including those in the retinoblastoma family (reviewed in [15]), but it was also dispensable for hHR23A binding (Figure 4D). Similarly, E2F1 proteins incapable of both cyclin A and retinoblastoma binding also formed complexes with hHR23A (Figure 4D). Of note, E2F1 contains 14 lysine residues that can potentially be modified by ubiquitylation and, given that the ubiquitin-binding UBA1 domain in hHR23A participates in the interaction between these two proteins, it is conceivable that no single region in E2F1 is indispensable.

### Inhibition of E2F1 degradation by hHR23 proteins

**Figure 5:**
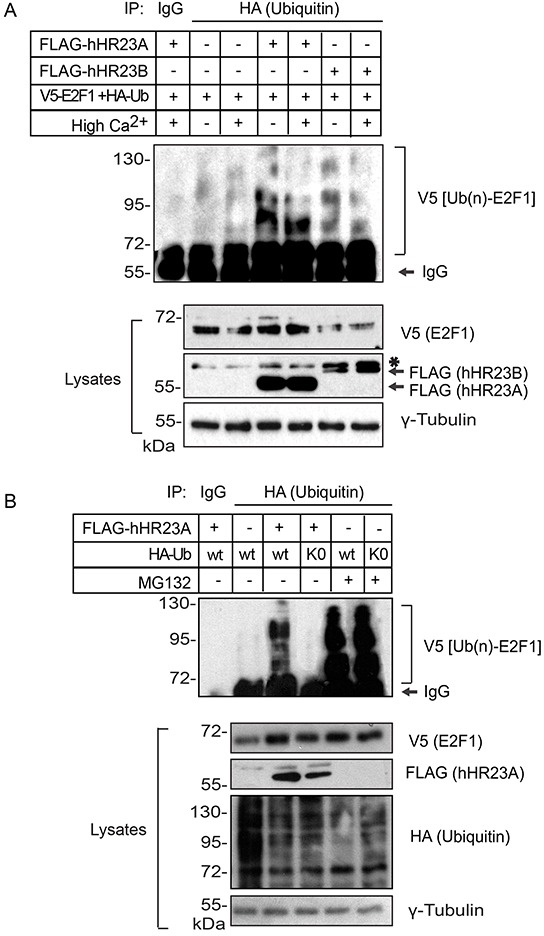
Modulation of ubiquitylated E2F1 degradation by hHR23 proteins **A.** Primary undifferentiated keratinocytes were transfected with vectors encoding V5-tagged E2F1 and HA-tagged ubiquitin, in the presence or absence of hHR23-encoding vectors, as indicated. The cells were cultured for 24 h in Low Ca^2+^ (undifferentiated keratinocytes) or High Ca^2+^ medium (differentiated keratinocytes) in the absence of MG132. Cell lysates were prepared and ubiquitylated proteins were immunoprecipitated with anti-HA antibodies. HA immune complexes were analyzed with anti-V5 antibodies, to detect ubiquitylated E2F1 in the immune complexes. The asterisk indicates a lower mobility non-specific band. **B.** Primary undifferentiated keratinocytes were transfected with a vector encoding V5-tagged E2F1 and either wild type (WT) ubiquitin, or a ubiquitin mutant lacking all Lys residues (K0), in the presence or absence of FLAG-tagged hHR23A. Keratinocytes were treated with vehicle (dimethylsulfoxide) or MG132 (10 μM) for 6 h, and cell lysates were prepared, immunoprecipitated with anti-HA antibodies and analyzed for the presence of ubiquitylated V5-E2F1, as in (A).

E2F1 ubiquitylation includes K11-, K48- and K63-linked polyubiquitin chains, which can promote its proteasomal degradation [16]. To begin to characterize the functional significance of the interactions between hHR23 proteins and E2F1, we exogenously expressed in keratinocytes V5-tagged E2F1 and HA-tagged wild type ubiquitin, in the presence or absence of hHR23 proteins. We then isolated HA-containing immune complexes and investigated therein the presence of E2F1. This approach allowed us to examine the effects of hHR23 on the abundance of ubiquitylated E2F1. In the absence of exogenous hHR23 proteins, and consistent with previous reports [17], we detected fairly low levels of ubiquitylated E2F1 in both undifferentiated and differentiated keratinocytes, likely because it efficiently undergoes proteasomal degradation (Figure 5A, 5B). The presence of either hHR23A or hHR23B substantially increased the abundance of ubiquitylated E2F1, irrespective of the differentiation status of the keratinocytes. Thus, hHR23 proteins appear to protect E2F1 from degradation, although this effect does not appear to involve interference with E2F1 ubiquitylation *per se* (Figure 5A, 5B). Next, we determined the effect of proteasomal inhibition on modulation by hHR23A of polyubiquitylated E2F1. To this end, we exogenously expressed V5-tagged E2F1 together with HA-tagged wild type ubiquitin and FLAG-tagged hHR23A as before, and assessed the effect of proteasomal inhibition by MG132 on the abundance of E2F1. We observed that the robust increase in polyubiquitylated E2F1 in the presence of hHR23A was slightly enhanced in MG132-treated cells (Figure 5B), indicating that exogenous expression of hHR23A does not impair proteasomal activity but interferes with the ability of the proteasome to target E2F1 for degradation.

The UBA domains of hHR23A exhibit maximum affinity for chains composed of 4-6 ubiquitin residues [18]. Cellular proteins can be modified by polyubiquitin chains linked through isopeptide bonds between the terminal glycine (G76) in ubiquitin and one of the seven Lys residues on another ubiquitin moiety. To determine whether the stabilizing effect of hHR32A required the presence of polyubiquitin chains on E2F1, we co-expressed V5-tagged E2F1, FLAG-tagged hHR23A and an HA-tagged mutant ubiquitin form lacking all lysine residues (K0). This mutant is presumably incapable of forming polyubiquitin chains, although it can still contribute to mono-ubiquitylation [19]. In the absence of MG132, we found negligible E2F1 immunoreactivity in ubiquitin-K0 immunoprecipitates (Figure 5B). Significantly, proteasomal inhibition was associated with the presence of abundant poly-ubiquitylated E2F1 species (Figure 5B). These observations suggest that hHR23A may preferentially modulate the degradation of poly-ubiquitylated E2F1, although mono-ubiquitin modification of the latter on one or more Lys residues likely also takes place in keratinocytes, without precluding its proteasomal degradation.

**Figure 6:**
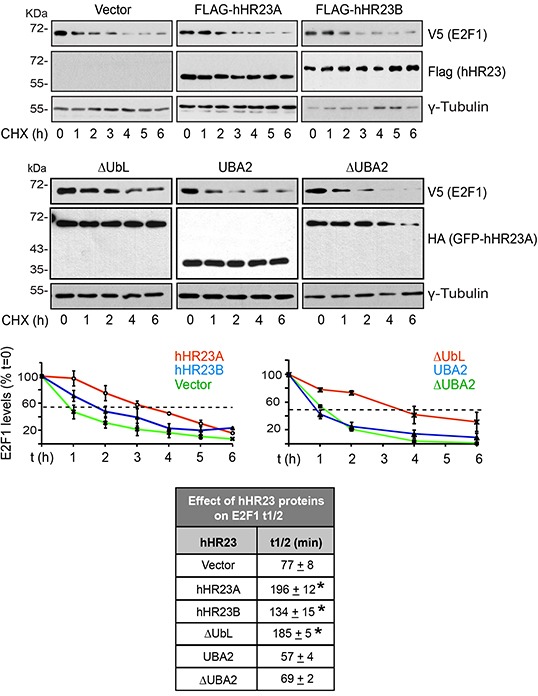
Modulation of E2F1 t½ by hHR23 proteins Primary undifferentiated keratinocytes were cotransfected with vectors encoding V5-tagged E2F1 and the indicated FLAG- or HA-tagged hHR23 proteins. Twenty-four hours after transfection, cycloheximide (CHX, 100 μg/ml, final) was added to the culture medium, and cell lysates were prepared at the indicated time intervals thereafter. The lysates were analyzed by immunoblot with the indicated antibodies, and γ-tubulin was used to normalize for protein loading. The graphs represent the mean ± SD of E2F1 levels quantified in replicate immunoblots generated from lysates prepared from three independent cell isolates, and were used to calculate E2F1 t½ (mean±SD, n=3) in the presence of each hHR23 protein, as summarized in the chart. The asterisks indicate P<0.05 relative to E2F1 t½ in the absence of any exogenous hHR23 protein (ANOVA).

The outcomes of hHR23 modulation of proteasome-mediated proteolysis are complex. In some cases, they deliver polyubiquitylated substrates to the proteasome for destruction [20]. In others, they impede the interaction of bound proteins with the proteasome, thus suppressing their degradation [21]. To further confirm that hHR23 negatively modulates E2F1 turnover, we used cycloheximide chase assays to determine E2F1 half-life (t½) in undifferentiated keratinocytes expressing wild type or mutant hHR23A proteins (Figure 6). The t½ of V5-E2F1 was 77±8 min, which was increased 3- and 2-fold, respectively, in the presence of similar levels of wild type hHR23A and hHR23B. Thus, although both hHR23 forms are able to stabilize E2F1, the effect of hHR23A would appear to be more pronounced. Similar to full-length hHR23A, ΔUbL GFP-hHR23A, which can efficiently bind E2F1, increased its t½ 3-fold to 185±5 min. In contrast, expression of UBA2 GFP-hHR23A did not significantly alter E2F1 t½, consistent with the notion that the stabilizing effect of hHR23A requires its association with E2F1, and that UBA2 does not associate with this transcription factor. Although the UBA2 domain does not participate in binding to or stabilizing E2F1, it is critical for the ability to hHR23A to escape proteasomal degradation itself, and its deletion markedly increases hHR23A turnover [22]. Consistent with these characteristics, we observed that, whereas the t½ of wild type hHR23A was > 6 h, the t ½ of ΔUBA2 GFP-hHR23A was only about 4 h (Figure 6). Further, ΔUBA2 GFP-hHR23A was without effect on E2F1 t½, suggesting that insufficient pools of the former protein may have been available to protect exogenous E2F1 from degradation.

### Modulation of DNA damage repair by hHR23A and E2F1

**Figure 7:**
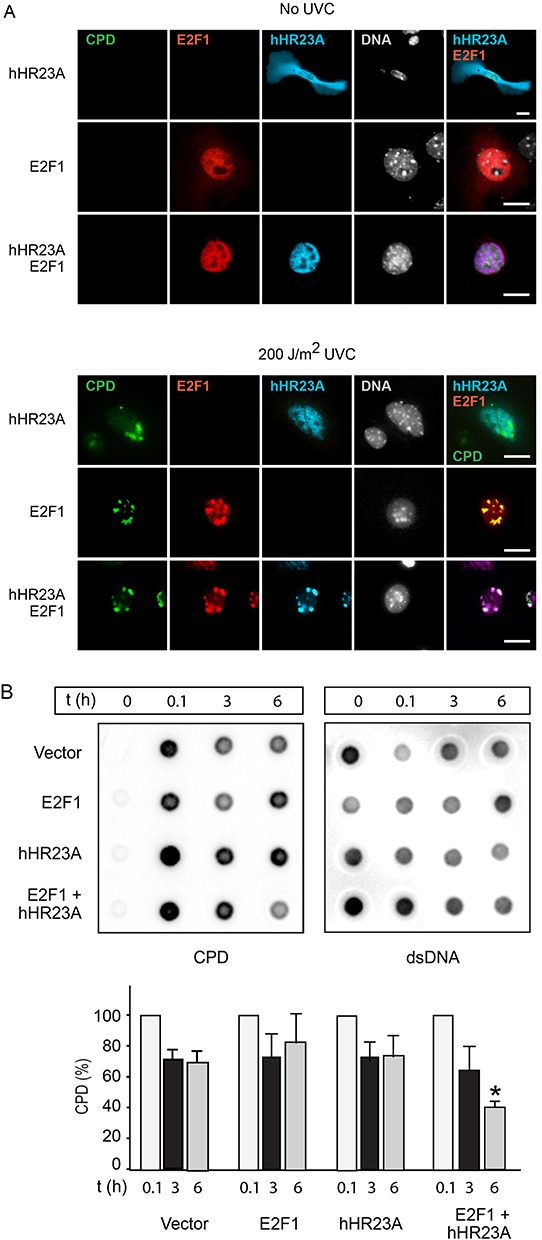
Increased repair of UV-induced DNA damage by E2F1 and hHR23A **A.** Keratinocytes were transfected with vectors encoding the indicated proteins, and 24 h later were subjected to UVC irradiation through 3-μm polycarbonate isopore filters, and cultured for 2 h. The cells were processed for immunofluorescence microscopy to detect cyclobutane pyrimidine dimers (CPD), V5-tagged E2F1 and HA-tagged hHR23A. Nuclear DNA was visualized with Hoescht 33342. Bar, 25 μm. **B.** HeLa cells transfected with plasmids encoding the indicated proteins were subjected to UVB irradiation (100 J/m^2^). Genomic DNA was isolated at the indicated times after UVB treatment, and analyzed for the presence of CPD as described in “Materials and Methods”. CPD levels were normalized to the amount of dsDNA. The histograms show the fraction of normalized CPD signal remaining after 3 or 6 h, relative to CPD 0.1 h after irradiation (set to 100%), for each transfection group. The results are expressed as the mean + SD (n=3), and the asterisks indicate P<0.05 (ANOVA).

Following DNA damage, hHR23A participates in NER processes, whereas E2F1 is stabilized, either promoting apoptosis or stimulating DNA repair [10]. Given that a major source of DNA damage in keratinocytes is UV radiation, we examined the responses of these two proteins to UV-induced DNA damage. We observed that endogenous E2F1 levels are increased by UVB radiation or by expression of hHR23A, and the presence of exogenous hHR23A further increased E2F1 levels in UVB-treated keratinocytes (Supplementary Figure S3). Next, we determined if hHR23A subcellular localization was regulated by DNA damage in primary keratinocytes, using filtered UV radiation coupled with immunofluorescence microscopy [12, 23]. Cells were irradiated with UVC light through a filter (3-μm pore size), and nuclear regions containing UV-induced photoproducts were identified with an antibody specific for CPD. Because the filters absorb about 90% of UV radiation, it was necessary to use light in the UVC spectrum, as it induces DNA damage more efficiently than UVB [23]. We observed that UV treatment of keratinocytes induced cytoplasm-to-nucleus translocation of hHR23A (Figure 7A). In the majority of cells expressing exogenous E2F1, we observed that E2F1 was substantially enriched in nuclear areas that exhibited CPD immunoreactivity (Figure 7A). Significantly, when E2F1 and hHR23A were co-expressed, both proteins concentrated in CPD-positive regions, indicating that E2F1 promotes enrichment of hHR23A at sites of UV-induced DNA damage in primary keratinocytes (Figure 7A).

We next investigated if the increased localization of hHR23A to DNA photolesions in the presence of E2F1 was associated with changes in DNA repair. To this end, we transfected cells with vectors encoding hHR23A and/or E2F1, subjected the cells to 100 J/m^2^ UVB radiation, and measured decreases in CPD abundance in genomic DNA (indicative of NER) as a function of time. Because UVB treatment of transfected primary keratinocytes resulted in variable proportions of transfected cell death among different cell isolates, we conducted this experiments on HeLa cervical carcinoma cells, in which no such variability was observed. CPD levels in control cells transfected with empty vector decreased by about 30%-40% of initial values 3 h after UVB treatment, and did not decrease substantially further 6 h after UVB treatment (Figure 7B). CPD levels in cells expressing E2F1 or hHR23A had decreased by about 20%-25% 6 h after UVB treatment. In contrast, in cells co-expressing E2F1 and hHR23A, CPD levels steadily decreased by about 40% and 60%, respectively, 3 h and 6 h following UVB exposure. Similar results were observed in RPMI 7951 human melanoma cells (data not shown). Thus, the joint expression of E2F1 and hHR23A accelerates CPD removal and DNA repair following UVB damage.

## DISCUSSION

**Figure 8:**
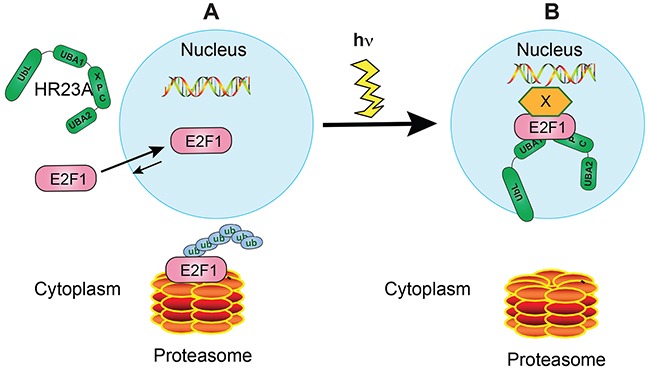
Proposed model for hHR23A and E2F1 regulation of DNA repair in undifferentiated keratinocytes **A.** In the absence of DNA damage, hHR23A remains mainly localized to the cytoplasm. E2F1 is known to undergo nucleocytoplasmic shuttling in these cells, with the equilibrium favouring nuclear concentration of this protein. Under these conditions, E2F1 turnover is promoted by polyubiquitylation and proteasomal degradation. **B.** DNA photodamage induces hHR23A nuclear translocation and E2F1 recruitment to sites of DNA lesions, with potential involvement of factors that serve as a bridge between DNA and E2F1. E2F1 can form complexes with hHR23A at those sites, promoting DNA repair. In a coordinate fashion, E2F1 is also protected from degradation through various mechanisms, likely including association with hHR23 proteins.

Our studies have uncovered a complex reciprocal regulation between hHR23 proteins and E2F1. Specifically, hHR23 proteins can bind E2F1 and protect it from proteasomal degradation. In turn, E2F1 can recruit hHR23A to the nucleus in the absence of DNA damage. Upon photodamage, E2F1 is stabilized and is recruited to DNA lesion sites, bringing hHR23A with it, and increasing the efficiency of DNA repair (Figure 8).

hHR23A and its yeast orthologue Rad23 function as shuttle proteins that deliver ubiquitylated cargo to the proteasome for proteolysis [24]. However, hHR23A can also associate directly with unmodified proteins. For example, the HIV-1 protein Vpr binds to the UBA2 and XPC domains of hHR23A [6]. Similarly, our *in vitro* binding assays with bacterially produced proteins demonstrate that hHR23A also binds unmodified E2F1. The association of hHR23A and E2F1 *in vivo* is likely more complex, due to the presence of ubiquitylated E2F1 pools normally targeted for proteasomal degradation in mammalian cells. Our domain mapping analysis clearly indicates that the UBA2 domain in hHR23A is neither necessary nor sufficient for E2F1 interaction. Our data also suggest that UBA1 and XPC are important regions involved in E2F1 binding in keratinocytes. Whereas the XPC domain may mediate binding to unmodified E2F1 residues, UBA1 may also contact ubiquitylated E2F1. An important area for future research will be to determine which domains in hHR23A and E2F1 bind to each other, independent of ubiquitin modifications.

The diversity of domains and possible modes of interaction of hHR23A and E2F1 imply that diverse cellular processes may be activated, depending on cell type, differentiation status and presence of DNA-related stresses. For example, under normal conditions, hHR23A appears to be largely cytoplasmic in undifferentiated keratinocytes. Significantly, E2F1 is capable of modulating hHR23A subcellular localization in these cells, as its exogenous expression results in nuclear concentration of hHR23A in the absence of DNA damage in these cells. Reciprocally, hHR23A is able to reduce E2F1 turnover in undifferentiated keratinocytes. The ability of E2F1 to modulate hHR23A subcellular localization is further emphasized by the observation that both E2F1 and hHR23A remain cytoplasmic as a result of differentiation in keratinocytes. E2F1 is indispensable in undifferentiated keratinocytes to maintain proliferative capacity and for their activation during epidermal repair after injury [25]. A potential role of hHR23A in these cells might be to stabilize E2F1 to ensure maintenance of a proliferative cell population. Further, it will be important to determine if hHR23A also plays a role in mediating the increases in E2F1 abundance observed in the regenerating epidermis. Similar stabilizing roles for hHR23A have been reported vis-à-vis p53 and ataxin-3 [8, 21].

Because of their location at the skin surface, epidermal keratinocytes are uniquely exposed to UV-related damage, and have evolved several protective mechanisms, including the ability of these cells to repair damaged DNA through nucleotide excision repair. The importance of these pathways for epidermal homeostasis is underlined by the existence of dermatological diseases such as xeroderma pigmentosum, which is associated with skin photosensitivity. In particular, individuals with this disorder exhibit hypersensitivity to solar UV light and frequent development of skin cancers [26]. Several genetic abnormalities that cause XP have been identified, including alterations in XPC. In response to DNA damage, various signaling proteins are recruited to areas with altered bases. Under these conditions, hHR23 proteins play key roles facilitating recognition by XPC of DNA lesion sites, contributing to the repair of transcriptionally silent genome regions or of non-transcribed strands of transcriptionally active genes [5, 7]. Given that E2F1 is most abundant in undifferentiated basal keratinocytes with proliferation potential [27], the cells of origin of nonmelanoma skin carcinoma, it is conceivable that a major role *in vivo* for the interaction between hHR23 proteins and E2F1 is as a protective mechanism against DNA damage and malignant transformation of undifferentiated keratinocytes with proliferative potential in the interfollicular epidermis and hair follicles.

The regulation of E2F1 in response to DNA damage is complex, depends on the damaging agent, and occurs through various mechanisms. The human orthologue of E2F1 is phosphorylated by the ATM and ATR kinases on Ser31 (Ser 29 in mouse E2F1) upon drug-induced DNA damage, promoting its binding to 14-3-3τ, which decreases its ubiquitylation and proteasomal degradation [28]. Under these circumstances, stabilization of E2F1 leads to the transcriptional activation of its pro-apoptotic targets *p73* and *Apaf1*. E2F1 can also be acetylated on several Lys residues in response to chemicals that cause DNA double-strand breaks, with similar outcomes on transcriptional activation of E2F pro-apoptotic targets [29, 30]. Efficient DNA repair in response to UV-induced DNA damage also requires E2F1, although different mechanisms appear to be involved. Under those conditions, E2F1 is also phosphorylated on Ser31 and stabilized [30, 31]. Phospho-E2F1 can bind Topoisomerase II b-binding protein 1 (TopBP1), decreasing *p73* and *Apaf1* transcription. TopBP1 also mediates recruitment of E2F1 to DNA double-strand breaks, where it colocalizes with BRCA1, and promotes NER through mechanisms that appear to involve formation of complexes containing TopBP1, E2F1 and GCN5 [11]. Indeed, E2F1-deficient cells are deficient in NER through mechanisms that involve impaired recruitment of DNA repair factors, such as GCN5, to sites of DNA damage. Further, a mouse E2F1 mutant protein lacking Ser29 is incapable of localizing to UV-induced sites of DNA damage, and epidermal keratinocytes expressing this protein exhibit increased susceptibility to UV-induced carcinogenic transformation [10]. Our data now show a novel mechanism that contributes to NER, through interactions between hHR23 proteins and E2F1, and which likely involves the recruitment of hHR23A by E2F1 to sites of DNA photodamage. Key issues for future research are to determine if E2F1-hHR23 complexes also contain XPC, and to establish the relative importance of the E2F1-hHR23 interaction to the overall capacity of keratinocytes to repair UV-induced DNA. This is a complex issue to address, given that individual depletion of hHR23 proteins or E2F1 impair cellular repair capacity. The identification of E2F1 mutants incapable of binding to hHR23A may provide answers to this question.

E2F1 is a well-established promoter of cell proliferation, but its roles in tumour formation and progression are complex, as it can either induce or suppress tumourigenesis. In the context of the epidermis, E2F1 is necessary for normal wound healing [25], and its overexpression leads to spontaneous tumour formation, exacerbated by the loss of p53 [32]. Paradoxically, E2F1 also protects the epidermis from UV-induced transformation, and cooperates with the retinoblastoma protein, pRB, to prevent spontaneous carcinogenic transformation of hair follicle keratinocytes through modulation of β-catenin/Wnt pathways [10, 33]. Our studies now provide new insights on the role of E2F1 in maintenance of genomic stability, underlining the importance of this transcription factor in maintenance of epidermal homeostasis.

## MATERIALS AND METHODS

### Reagents and antibodies

Cholera toxin (100) and insulin (16634) were from List Biological (Campbell, CA, USA) and Invitrogen (Carlsbad, CA, USA), respectively. Polyethyleneimine (PEI; 25 kDa linear (23966) was from Polysciences (Warrington, PA). Chelex 100 resin was purchased from Biorad (142-2832; Mississauga, ON, Canada). All other reagents were purchased from Sigma (St. Louis, MO, USA). The antibodies used were: anti-V5 (R960-25; Invitrogen, Carlsbad, CA, or ab9113, Abcam, Cambridge, MA), and anti-E2F1 (TA308764; OriGene, Rockville, MD). Antibodies against cyclobutane pyrimidine dimers (CPD) were from CosmoBio Co. (NMDND001, clone TMD-2, Tokyo, Japan); anti-double strand (ds) DNA (ab27156, Abcam, Cambridge, MA); anti-HA (Y-11) and anti-Rad23A (D-6) antibodies were purchased from Santa Cruz (Santa Cruz, CA), anti-GAPDH was from Enzo Life Sciences (ADI CSA 335, Farmingdale, NY); anti-FLAG M2 (F1804) and anti-γ-tubulin (T6557) were from Sigma (St. Louis, MO). Horseradish peroxidase-conjugated goat anti-mouse (115–035-146) and anti-rabbit (111-035-144) antibodies were from Jackson Immuno Research Laboratories (West Grove, PA). Alexa Fluor®-conjugated phalloidin (A12379), goat anti-rabbit and goat anti-mouse IgG were from Molecular Probes/Invitrogen (Eugene, OR).

### Plasmids

The vectors encoding V5-tagged wild type and mutant human E2F1 proteins have been described [13, 14, 34]. Vectors encoding the FLAG- and HA-tagged hHR23A, hHR23B and their truncation mutants ΔUbL, ΔUBA2, and UBA2 [8] were generous gifts from Dr. Christine Blattner (Institute of Toxicology and Genetics, Karlsruher Institut für Technologie, Germany). The vectors encoding wild type HA-tagged ubiquitin and GST-E2F1 were kind gifts, respectively, from Dr. David Litchfield (Western University) and Dr. Paul Hamel (University of Toronto). The plasmids pRK5-HA-Ubiquitin-K0 (missing all lysine residues; Addgene plasmid 17603) and pRK5-HA-Ubiquitin-WT (Addgene plasmid 17608) were obtained from Addgene (Cambridge MA), where they were deposited by Drs. Ted Dawson and Cecile Pickart. The bacterial expression vector encoding Flag-tagged hHR23A was generated by polymerase chain reaction (PCR) and cloned into the HindIII and NotI sites of pET32b-TRX. Mammalian expression vectors encoding GFP- and HA-tagged hHR23A and its deletion mutants ΔUbL, ΔUBA1, ΔUBA2, ΔXPC, XPC-UBA2, UBA1 and UBA2 were generated by PCR amplification and were verified by dideoxy sequencing.

### Cell culture and transfection

All animal experiments were approved by the University of Western Ontario Animal Care Committee (Protocol No. 2015-021), in accordance with regulations and guidelines from the Canadian Council on Animal Care. Primary mouse keratinocytes were isolated from 2 d-old CD-1 mice and cultured in Ca^2+^-free EMEM (06-174G, Lonza, Rockland, ME) supplemented with growth additives and 8% fetal bovine serum (FBS) pre-treated with Chelex resin, as described [17, 34]. Keratinocyte differentiation was induced by culture in growth medium containing 1 mM CaCl_2_ (“High-Ca^2+^ medium”) or with 0.12 mM CaCl_2_, as noted in individual experiments, for at least 24 h. HeLa and RPMI-7951 human melanoma cells were purchased from the American Type Culture Collection, and cultured in HyQ Reduced Serum DMEM (SH30565.01, Hyclone, Logan, UT), supplemented with 4% FBS. Cells were transfected with PEI, as described [35]. Where indicated, transfected keratinocytes were induced to differentiate by a 24h culture period in High Ca^2+^ medium, initiated 4h after transfection. For E2F1 turnover experiments, cycloheximide (CHX, 100 μg/ml, final) was added to keratinocyte cultures 24 h after transfection; cell lysates were prepared at timed intervals after CHX addition and analyzed by immunoblot. For experiments involving proteasomal inhibition, cells were treated with MG132 (10 μM) for 6 h prior to harvesting.

### RNA isolation and qPCR

Total RNA was isolated from cultured cells or from tissues harvested from 2 d-old CD-1 mice, using RNeasy minikits (Qiagen, Louisville, KY), following the manufacturer's instructions. Epidermis and dermis samples were obtained from skin treated with 8 mg/ml dispase (04 942 078 001, Roche Diagnostics, Indianapolis, IN) for 15 minutes at 37°C, as described [36]. RNA concentration and quality were determined with an Agilent 2100 Bioanalyzer (Agilent, Santa Clara, CA). Only samples with RNA integrity numbers ≥8.5 were used. RNA (200 ng/sample) was reverse-transcribed using SuperScript II RT (Invitrogen, Carlsbad, CA), as per manufacturer's protocol. qPCR reactions were conducted on a CFX384 Real-Time PCR system (Bio-Rad) operated by CFX Manager software (version 1.6). cDNA samples corresponding to 20 ng of RNA were amplified using PerfeCTa qPCR SuperMix (Quanta BioSciences, Gaithersburg, MD) and the following primers (400 nM, final): mHR23A forward 5′-AAGATCCGCATGGAACCTGA-3′ and reverse 5′-TGGTCACCATGACAACCACAA-3′; mHR23B forward 5′-CTTAGGCACCATGCAGGTCA-3′ and reverse 5′-CGGAAAGGCATCTTTCCCCT-3′. The results were normalized to the expression of the housekeeping genes *Rpl16* and *Rps29*, which encode ribosomal protein L16 and S29, respectively [37]. Replicate cDNA samples were amplified for 40 cycles (98°C for 10 sec, and 58°C for 30 seconds per cycle). Primer specificity and amplicon sizes were confirmed, respectively, by analysis of melt curves conducted between 65°C and 95°C, in 0.5°C increments, and agarose gel electrophoresis. Relative *mHR23A* and *mHR23B* mRNA levels were calculated using the ΔΔCt method. At least three RNA preparations obtained from three different cell isolates were analyzed.

### Immunoblot analysis and immunoprecipitation

Cell lysates were prepared using modified RIPA-lysis buffer (50 mM Tris-HCl pH 7.4, 150 mM NaCl, 0.1% NP-40, 0.5% sodium deoxycholate, 0.1% sodium dodecylsulphate (SDS), 1 mM Na_3_VO_4_, 5 mM NaF, 1 mM phenylmethylsulfonyl fluoride, 1 μg/ml each aprotinin, leupeptin and pepstatin). In experiments assessing E2F1 ubiquitylation, the lysis buffer also contained 25 mM N-ethylmaleimide (NEM). Proteins in lysates (30 μg/sample) were resolved by denaturing polyacrylamide gel electrophoresis and transferred to polyvinylidene difluoride membranes, which were probed with antibodies indicated in individual experiments. After antibody probing, protein signals were visualized by treating the membranes with Amersham ECL Prime Western Blotting Detection Reagent (GE Healthcare Life Sciences, Mississauga, Canada). For immunoprecipitation assays, cell lysates prepared as described above (1.5 mg protein/sample) were incubated with antibodies indicated in individual experiments (3 μg/sample) for 16 h at 4°C. The immune complexes were captured by incubation with protein A/G magnetic beads (88802, Pierce Biotechnology/Thermo Scientific, Rockford, IL) for 1 h at 22°C, followed by 5 washes with PBS containing 0.05% Tween-20, and elution in 1x Laemmli sample buffer for 10 min at 25°C. Immune complexes were resolved and analysed by immunoblot, as described above. The results shown in figures are representative of 3-8 experiments, each conducted with independent cell isolates.

### Indirect immunofluorescence microscopy

Twenty-four hours after transfection, cells were fixed and processed for microscopy, as described [14]. The fraction of cells with nuclear or cytoplasmic E2F1 or hHR23A was determined from the analysis of ≥100 cells per experiment. To visualize cyclobutane pyrimidine dimers (CPD), cells were fixed in freshly diluted 4% paraformaldehyde in PBS, followed by incubation in 0.2 N HCl for 30 min at 22°C, to denature nuclear DNA. The cells were washed 5 times with PBS and incubated with primary antibodies, as described [14]. All photomicrographs shown are representative of experiments conducted on duplicate samples at least thrice, and scoring ≥ 100 transfected cells per experiment. Images were obtained with a Leica DMIRB fluorescence microscope equipped with an Orca-ER digital camera (Hamamatsu Photonics, Hamamatsu City, Japan), using Volocity 6.1.1 software (Improvision-PerkinElmer, Waltham, MA). The results shown in figures are representative of 3-8 experiments each conducted with independent cell isolates.

### UV damage and repair assays

The co-localization of proteins to sites of UVC radiation-induced DNA damage was conducted as described [11, 12], with minor modifications. Keratinocyte monolayers were rinsed once with PBS, leaving a thin layer of buffer. A sterile 3-μm pore polycarbonate membrane (TSTP02500, EMD Millipore Corp. Etobicoke, Canada) was gently placed onto the cells, followed by irradiation with 254 nm UVC light (200 J/m^2^), using a Bio-Rad GS Gene Linked UV Chamber. The membrane was removed. The cells were cultured in normal growth medium for 2 h and processed for immunofluorescence microscopy.

Repair of UV-induced DNA damage was assessed as described [11], with some modifications. HeLa or RPMI-7951 cells seeded in 10-cm culture dishes were transfected with vectors encoding E2F1 and/or hHR23A. Sixteen hours after transfection, the cells were trypsinized, re-seeded in quadruplicate 35-mm culture dishes, and cultured an additional 16-h interval to allow attachment. Duplicate samples were then mock-irradiated or subjected to UVB treatment (302 nm, 100 J/m^2^), using a UVM-28 EL series 8-watt lamp (UVP, Upland, CA). At timed intervals following UVB irradiation, genomic DNA was purified with DNeasy Blood & Tissue kits (Qiagen, Toronto, Canada) and denatured by boiling at 95°C for 5 minutes. Replicate 200-ng samples were spotted onto Hybond N^+^ membranes (Amersham, NJ), which were baked at 80°C for 1 h, and placed onto filter paper pre-wetted with 0.4 N NaOH for 20 min. The membranes were blocked for 16 h at 4°C in 5% (w/v) non-fat milk diluted in Tris-buffered saline supplemented with 0.2% Tween-20 (TBST). After two TBST washes, membranes were incubated for 2 h at 22°C with anti-CPD antibodies (1:5000 (v/v) dilution in TBST). The membranes were re-probed with anti-dsDNA antibodies, to normalize for DNA content. CPD and dsDNA signals were visualized with a VersaDoc-3000 imager (Bio-Rad Laboratories) and quantified with Quantity One software (Bio-Rad Laboratories). All results shown are representative of at least three independent experiments. Statistical analyses were conducted using ANOVA, and significance was set at P<0.05.

## SUPPLEMENTARY FIGURES



## Abbreviations

CPD: cyclobutane pyrimidine dimers
GAPDH: glyceraldehyde 3-phosphodehydrogenase
GFP: green fluorescent protein
GST: glutathione
HA: hemagglutinin
NER: nucleotide excision repair
PCR: polymerase chain reaction
qPCR: reverse transcription-quantitative polymerase chain reaction
t½: half-time
XP: Xeroderma pigmentosum
XPC: Xeroderma pigmentosum complementation group C
