# Supplementary material for: E2F1 interactions with hHR23A inhibit its degradation and promote DNA repair
Source: Oncotarget. 2016 Mar 25;7(18):26275–92. doi: 10.18632/oncotarget.8362 (PMC5041980; doi:10.18632/oncotarget.8362)
Supplement: Supplementary file 1 [file oncotarget-07-26275-s001.pdf]

SUPPLEMENTARY FIGURES

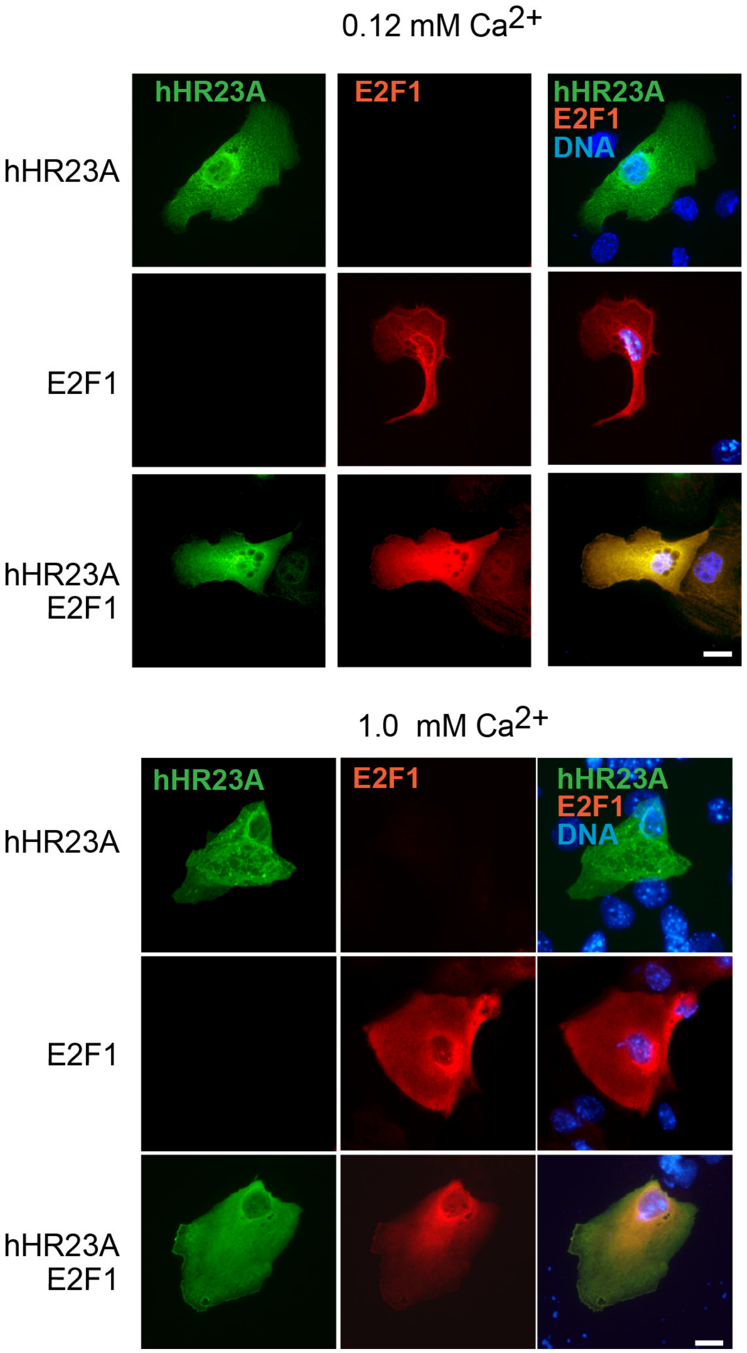

Supplementary Figure S1: Subcelllar distribution of hHR23A and E2F1 in differentiated keratinocytes. Keratinocytes were transfected with vectors encoding the indicated proteins, and 4 hours after transfection were induced to differentiate by culture in growth medium with the indicated Ca^{2+} concentration. The cells were processed for immunofluorescence microscopy using anti-V5 and anti-HA antibodies to detect, respectively, E2F1 and hHR23A. DNA was visualized with Hoescht 33342. Bar, 25 µm.

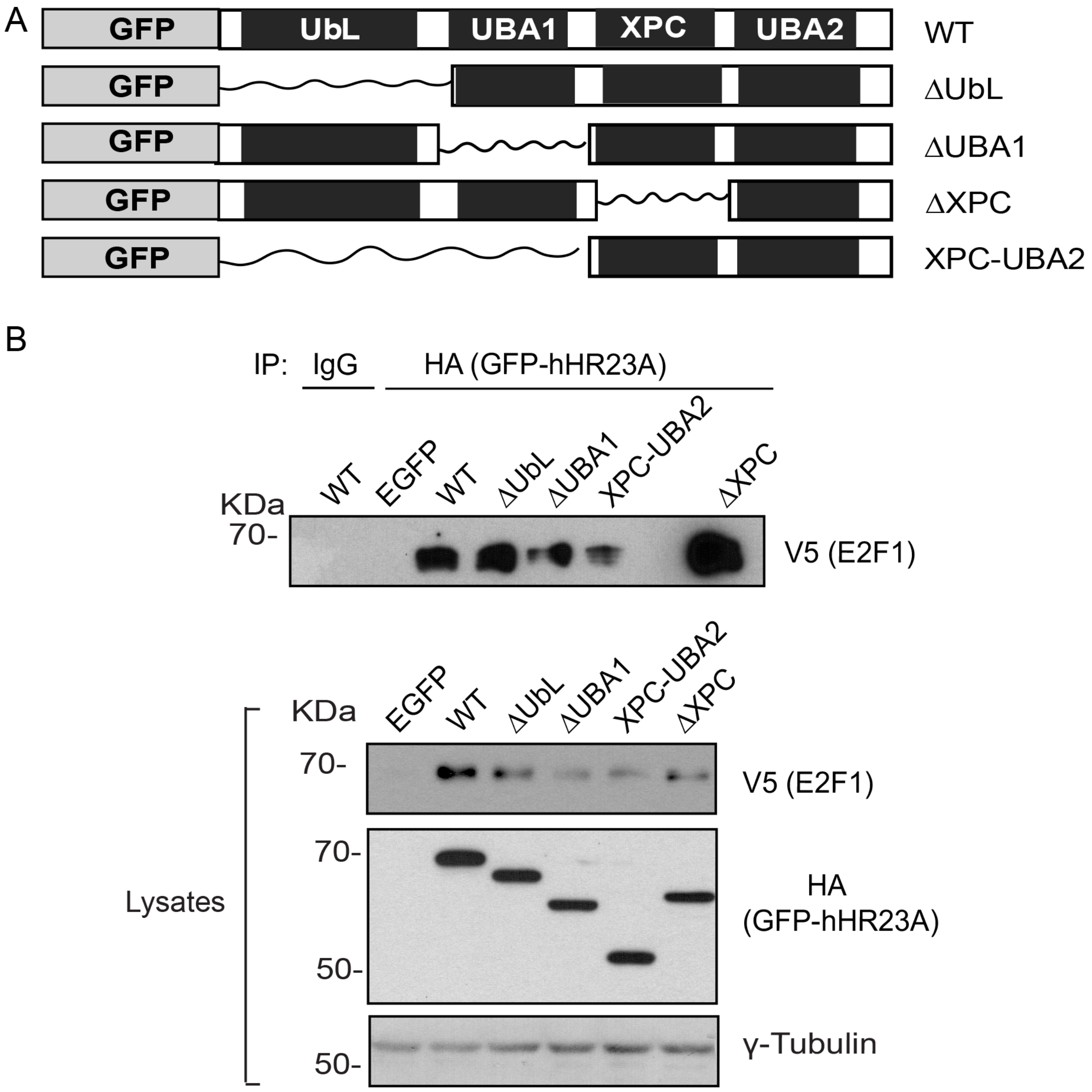

Supplementary Figure S2: The XPC domain in hHR23A is dispensable for binding to E2F1. A. Schematic of GFP- and HA-tagged hHR23A proteins tested for their ability to bind E2F1. **B.** Primary keratinocytes were transfected with vectors encoding the hHR23A proteins indicated, as well as V5-tagged E2F1. Twenty-four hours after transfection, cell lysates were prepared and hHR23A immune complexes were isolated using anti-HA antibodies. The immune complexes were resolved by denaturing gel electrophoresis, and analyzed with the indicated antibodies. γ-Tubulin was used to normalize for protein loading. Lysates from cells co-expressing E2F1 and wild type (WT) hHR23A were used for immunoprecipitations using anti-HA antibodies, or an unrelated, control IgG.

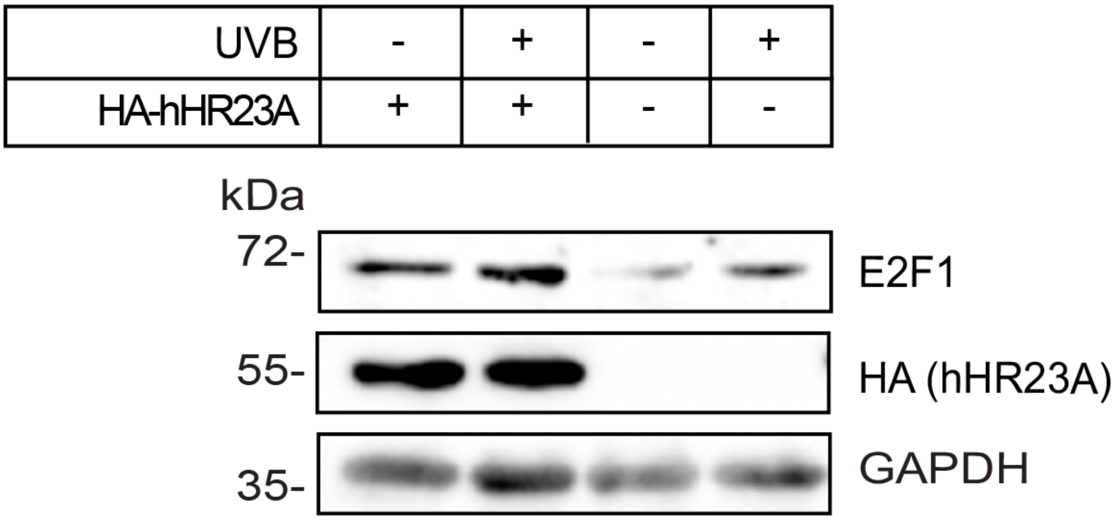

Supplementary Figure S3: Effect of UVB radiation on E2F1 levels in keratinocytes. Primary keratinocytes expressing HA-tagged hHR23A were exposed to 250 J/m2 of UVB, and whole cell lysates were prepared 24 h after irradiation to analyze levels of the indicated proteins. Glyceraldehyde 3-phosphodehydrogenase (GAPDH) levels were used to normalize for protein loading.
